# Adenosine deaminase deficiency: a review

**DOI:** 10.1186/s13023-018-0807-5

**Published:** 2018-04-24

**Authors:** Aisling M. Flinn, Andrew R. Gennery

**Affiliations:** 1 Institute of Cellular Medicine, Newcastle University, Newcastle upon Tyne, UK; 2 Great North Children’s Hospital, Clinical Resource Building, Floor 4, Block 2, Queen Victoria Road, NE1 4LP, Newcastle upon Tyne, UK

**Keywords:** Adenosine deaminase, Severe combined immunodeficiency, Neurodevelopment, Haematopoietic stem cell transplantation, Gene therapy, Pulmonary alveolar proteinosisis

## Abstract

Adenosine deaminase (ADA) deficiency leads to an accumulation of toxic purine degradation by-products, most potently affecting lymphocytes, leading to adenosine deaminase-deficient severe combined immunodeficiency. Whilst most notable affects are on lymphocytes, other manifestations include skeletal abnormalities, neurodevelopmental affects and pulmonary manifestations associated with pulmonary-alveolar proteinosis. Affected patients present in early infancy, usually with persistent infection, or with pulmonary insufficiency. Three treatment options are currently available. Initial treatment with enzyme replacement therapy may alleviate acute symptoms and enable partial immunological reconstitution, but treatment is life-long, immune reconstitution is incomplete, and the reconstituted immune system may nullify the effects of the enzyme replacement. Hematopoietic stem cell transplant has long been established as the treatment of choice, particularly where a matched sibling or well matched unrelated donor is available. More recently, the use of gene addition techniques to correct the genetic defect in autologous haematopoietic stem cells treatment has demonstrated immunological and clinical efficacy. This article reviews the biology, clinical presentation, diagnosis and treatment of ADA-deficiency.

## Background

Adenosine deaminase (ADA) is a key enzyme of the purine salvage pathways and deficiency caused by mutations in the *ADA* gene results in one of the more common causes of autosomal recessive severe combined immunodeficiency (SCID), accounting for approximately 10–15% of cases in outbred populations [1]. Absent or impaired ADA function leads to the accumulation of the toxic metabolites adenosine, 2’deoxyadenosine and deoxyadenosine triphosphate (dATP). ADA-deficient SCID is characterized by severe lymphocytopaenia affecting T-and B-lymphocytes and NK cells, but, because of the ubiquitous nature of the enzyme, non-immunological manifestations are also observed, including neurodevelopmental deficits, sensorineural deafness and skeletal abnormalities. The incidence of ADA-deficiency in Europe is estimated to be between 1:375,000 to 1:660,000 live births [2]. Early diagnosis of ADA-deficient SCID and initiation of treatment is essential in this otherwise fatal condition. Current treatment options include enzyme replacement therapy (ERT), allogeneic haematopoietic stem cell transplant (HSCT), and autologous gene therapy (GT).

### Biochemistry

ADA is a ubiquitously expressed metabolic enzyme, although level of enzyme activity varies, with highest levels observed in lymphoid tissues, particularly the thymus, the brain and gastrointestinal tract [2], and is expressed both intracellularly and on the cell surface complexed with CD26 [3]. With purine nucleoside phosphorylase, it forms an essential component of the purine salvage pathway, responsible for the irreversible deamination of adenosine and 2’deoxyadenosine into inosine and 2’deoxyinosine respectively. Absent or impaired function consequently results in both intracellular and extracellular accumulation of these substrates. Adenosine primarily derives from breakdown of adenosine triphosphate (ATP) and RNA, and 2’deoxyadenosine from breakdown of DNA. 2’deoxyadenosine irreversibly inhibits the enzyme S-adenosylhomocysteine (SAH) hydrolase causing accumulation of SAH, which subsequently prevents S-adenosylmethionine-mediated methylation processes required for normal thymocyte differentiation, likely contributing to the impairment of T-lymphocyte development evident in ADA-deficiency [4]. Increased intracellular uptake of 2’deoxyadenosine followed by phosphorylation by deoxycytidine kinase leads to accumulation of deoxyadenosine triphosphate (dATP) which inhibits ribonucleotide reductase, preventing normal DNA synthesis and repair [5]. Adenosine is an important extracellular signalling molecule; disruption of these signalling pathways is thought to interfere with normal immune responses [6]. Adenosine receptors belong to the family of G protein-coupled receptors, of which there are four subtypes (A_1_, A_2A_, A_2B_ and A_3_), which play different roles in regulating normal cellular physiology in a wide variety of tissues including the brain, cardiovascular system and lungs [7].

### Diagnosis

Diagnosis of ADA-deficiency is established by biochemical and molecular genetic testing. Biochemical testing demonstrates absent or greatly reduced ADA activity (< 1% of normal) and marked elevation of the metabolite dATP or total dAdo nucleotides (the sum of dAMP, dADP and dATP) in erythrocytes. Reduced activity of SAH hydrolase in erythrocytes (< 5% of normal) is also characteristic [8]. If a patient with suspected ADA-deficiency has had a recent blood transfusion, analysis of ADA activity can be measured in the parents, with reduced activity seen in heterozygous carriers, or can be performed on non-erythroid cells such as leukocytes. Fibroblasts can also be used, but fibroblast cultures are usually not readily available and this may delay diagnosis. Molecular genetic diagnosis relies on the identification of bi-allelic pathogenic mutations in the *ADA* gene, located on chromosome 20q12-q13.11 and in which over 70 causative mutations have been identified.

Supportive laboratory findings include lymphocytopaenia, with absence of T- and B-lymphocytes and NK cells and low serum immunoglobulins, although in early infancy IgG may be normal due to materno-placental transfer. T-lymphocyte proliferative responses are low or absent, as are specific antibody responses. The level of metabolic substrates and the genotype have been shown to correlate with the severity of the clinical phenotype [9].

### Clinical manifestations

#### Immune-system - effects on a cellular level

The dominant consequences of ADA deficiency are on the immune system, causing severe depletion of T- and B-lymphocytes and NK cells, resulting in impaired cellular and humoral immunity. High levels of ADA are expressed in lymphoid tissues due to the high levels of cell turnover, particularly in the thymus, likely accounting for the resulting severe lymphocytotoxic effects of deficiency [10]. The underlying mechanisms responsible for the deleterious effects on the immune system have been elucidated with the use of ADA-deficient experimental models. There are pronounced effects on thymocyte development, although the precise stage at which this occurs is unknown. Apasov et al. demonstrated extensive apoptosis in the thymi of ADA(−/−) murine models but not in the peripheral lymph nodes and spleen, demonstrating the detrimental effect on developing thymocytes. Apoptosis in the thymi was evident predominantly at the cortico-medullary junction and particularly affected double positive thymocytes. Peripheral T-lymphocytes were also abnormal, with aberrant distribution in secondary lymphoid tissues and expression of cell markers, as well as defective T-lymphocyte signaling through the TCR [11]. It is thought that the combination of both intracellular accumulation of toxic substrates and defective T-lymphocyte signaling contribute to the depletion of developing thymocytes.

The B-lymphocyte compartment is also affected in ADA-deficiency with patients exhibiting severe B-lymphocytopaenia and hypogammaglobulinaemia, although, in contrast to T-lymphocytes, early B-lymphocyte development does not appear to be disturbed [12]. Abnormal splenic germinal centre architecture is observed suggesting impaired antigen-dependent B-lymphocyte maturation, and B-lymphocytes also displayed reduced proliferative abilities, increased apoptosis and impaired signaling upon activation [12]. This suggests that the B-lymphocyte defect is more likely to be related to impaired differentiation due to an intrinsic defect rather than solely due lack of appropriate CD4+ T-lymphocyte help. Impaired V(D)J recombination due to increased dATP levels may also negatively affect B-lymphocyte diversity and function [13].

#### Immune system - clinical manifestations

As a result of severely defected cellular and humoral immunity, the typical presentation of ADA-deficiency occurs early in life with severe infections and failure to thrive, and affected individuals will normally succumb within the first or second year of life without intervention. The clinical picture of ADA-deficient SCID is similar to other genetic forms of SCID, with persistent diarrhoea, dermatitis, and serious infections, often caused by opportunistic pathogens such as *Pneumocystis jiroveci,* being characteristic. Physical findings include absent thymus gland on thoracic-radiographs and absence of lymphoid tissues.

#### Non-immune manifestations

The ubiquitous nature of ADA also means that consequences of deficiency are not limited to lymphocytes, and many other systemic non-immunological features are also observed, with known impact on the nervous, auditory, skeletal, pulmonary, hepatic and renal systems as well as cognitive and behavioural abnormalities. Non-immunological manifestations have become more apparent in recent years as survival and immune reconstitution improves following stem cell transplantation, and awareness and identification of involvement of multiple organ systems is essential to allow timely optimal management.

Children with ADA-deficiency have been shown to exhibit a range of behaviour abnormalities, including attention deficits, hyperactivity, aggression and social problems, which appear to develop independently of the influences associated with HSCT [14, 15]. IQ levels are lower in children with ADA-deficient SCID compared to the population average and compared to children with other forms of SCID [15]. High levels of ADA expression found in the brain [10], and the finding that the total IQ scores correlate with the level of dATP at diagnosis [14], further support the theory that cognitive impairment is both a consequence of the metabolic disturbance in ADA-deficiency and dependent on the degree of deficiency.

Bilateral sensorineural hearing loss was first reported in two patients with ADA-deficiency who had been successfully treated with HSCT. Structural and infectious causes were excluded and both patients did not receive any conditioning prior to HSCT, precluding that as a potential cause and implicating the underlying metabolic defect [16]. A high prevalence of bilateral sensorineural hearing loss (58%) was reported in a cohort of 12 patients with ADA-deficiency who had been treated with HSCT [17]. In this study, no relationship was found between deafness and dATP levels.

The metabolic role of adenosine deaminase and consequences of toxic substrate accumulation in the lungs has been demonstrated in experimental models, with ADA(−/−)mice displaying severe pulmonary inflammation, with accumulation of activated macrophages and eosinophils, and airway remodeling, reversible upon initiation of ERT [5]. Mouse models have also shown that prolonged exposure to high concentrations of adenosine in the lung due to treatment with low dose ERT leads to development of pulmonary fibrosis, but these changes were reversed upon reducing pulmonary adenosine levels [18]. In ADA-deficient patients, similar pulmonary manifestations are seen, and non-infectious pulmonary disease, including pneumonitis and pulmonary alveolar proteinosisis (PAP), is found more frequently than in other genetic forms of SCID [19]. 43.8% of patients with ADA-deficient SCID had PAP in one study that rapidly resolved (in all but one patient) following commencement of ERT [20].

Skeletal abnormalities such as involving the costochondral joints are widely reported, possibly related to an imbalance between nuclear factor-κB ligand (RANKL) and osteoprotegerin (OPG), disturbing the interaction between osteoblasts and osteoclasts and subsequent bone formation, although abnormalities are mainly only apparent on radiological imaging without dysmorphic consequences [21–24]. The effect of toxic metabolites on bone marrow may play a role in the ‘auto-conditioning’ evident in ADA-deficient SCID, with the creation of stem cell niches, facilitating donor hematopoietic stem cell engraftment. However, skeletal abnormalities have also been reported in other immunodeficiencies and complete correction following therapy is not seen suggesting other factors involved in the pathogenesis.

Hepatic involvement in ADA-deficiency appears to differ between mice and humans. Murine ADA(−/−) models display severe hepatocellular degeneration that is fatal in the perinatal period [25]. In comparison, a severe degree of hepatic impairment is not normally seen in ADA-deficient patients, although there is a case report of a patient with ADA-deficient SCID who developed rapid fatal hepatic failure which could not be attributed to infection [26], and a neonate with ADA-deficient SCID with hepatitis and hyperbilirubinaemia which resolved with ERT [27]. Reports of renal involvement in ADA-deficiency include the occurrence of mesangial sclerosis found in 7/8 autopsies of ADA-deficient patients, with 6/8 also demonstrating cortical sclerosis of the adrenal glands [22]. Atypical haemolytic uraemic syndrome was reported in 4 patients with ADA-deficiency, 2 who recovered with mild or no residual renal impairment following supportive management and initiation of ERT [28]. Dermatofibrosarcoma protuberans is a rare malignant skin tumour, which has been reported to occur with greater frequency in patients with ADA-deficiency, but the mechanism behind this is unclear [29].

While ADA-deficiency is widely accepted as a systemic metabolic disorder, it is important to consider that certain systemic manifestations have only been reported in a small number of patients. Other contributing factors such as infectious agents may be involved and further investigation into the underlying pathogenesis of these manifestations is needed. Nevertheless, awareness of multi-organ involvement is essential for optimal patient care.

### Partial and late onset ADA-deficiency

There is heterogeneity in the phenotype of ADA-deficiency, with approximately 15–20% of patients exhibiting a ‘delayed clinical onset’ presenting with less severe, but gradually worsening, combined immune deficiency later in life, usually within the first decade, but occasionally in adulthood [8, 30, 31]. Clinical manifestations in this ‘delayed onset’ group include recurrent, but less severe infections, particularly affecting the sinopulmonary tract. Viral infections with papilloma virus also occur [32]. Autoimmunity, allergy and elevated IgE levels can also occur [2]. Because of this spectrum of clinical phenotypes it is important to consider the diagnosis of ADA-deficiency in older individuals, as delay in recognition leads to deterioration in immunological function and the development of irreversible sequelae of recurrent and chronic infections. Screening has also identified asymptomatic individuals who have very low or absent ADA activity in erythrocytes, but greater levels of ADA activity (2%–50% of normal) in nucleated cells, so called ‘partial ADA-deficiency’ [33–37]. These patients have seemingly normal immune function and life expectancy, although long-term follow-up data is currently unavailable to confirm this.

### Management

Unlike other forms of SCID, management of ADA-deficiency includes multiple options; ERT, allogeneic HSCT, and autologous GT, of which only the latter two are curative.

ERT with polyethylene glycol-conjugated adenosine deaminase (PEG-ADA) is the one therapeutic option that is not definitive in terms of disease correction but allows systemic clearance or ‘detoxification’ of the toxic metabolic substrates. ERT is an option if there is not a suitable HSCT donor, or if there are contraindications to HSCT, however, long-term ERT is associated with sub-optimal immune reconstitution [38]. Other limitations to ERT include lack of availability in some countries, high cost and the fact that lifelong treatment is required. It is also a short term option used as a stabilizing bridge to HSCT or GT to improve endogenous immune function and help in recovery from infections or in the setting of pulmonary alveolar proteinosis to optimise clinical condition prior to definitive therapy [39]. Use of ERT and timing of cessation prior to allogeneic HSCT must be carefully considered as improving recipient immunity poses a potentially increased risk of graft rejection, but cessation of ERT subjects the patient to a significantly increased risk of infection. Interestingly, Hassan et al. showed no difference in survival outcome between patients who did and did not receive ERT ≥ 3 months prior to HSCT, but the majority of the group who received ERT proceeded to have matched unrelated donor/mismatched unrelated donor (MUD/MMUD) or haploidentical donor transplants [40]. ERT may be continued for one month following GT, or up to the time of infusion, to maintain low levels of toxic metabolites to facilitate engraftment of the gene-corrected cells [39].

Traditionally, HSCT has been the treatment of choice for ADA-deficient SCID, usually performed as soon as possible following diagnosis to minimise the time exposed to high levels of toxic metabolites and before acquisition of infections. In the largest study to date examining the outcome of 106 patients with ADA-deficient SCID following HSCT, earlier HSCT was associated with a better overall survival but this did not reach statistical significance, possibly due to smaller patient numbers in the older groups [40]. A superior overall outcome is observed following HSCT using matched sibling and family donors (MSD/MFD) compared to MUD and haploidentical donors (86% and 81% versus 66% and 43% respectively) [40]. This may be related to faster availability of sibling or family donors, likely resulting in a better clinical condition going into HSCT. MSD and MFD HSCTs are also usually performed without serotherapy, impacting positively on the rate of T-lymphocyte recovery and clearance of viral infections in these patients. Outcome is also significantly improved in non-conditioned HSCT compared to myeloablative conditioning, although lack of conditioning may also impair engraftment, particularly with haploidentical donors [40]. Unconditioned HSCT using a MSD or MFD is associated with successful cellular and humoral immune reconstitution, although long-term outcome of immune status is unknown and further follow-up is necessary [40]. This is in contradisctinction to other forms of SCID, and it may be that the local toxic effects of ADA deficiency on the marrow act as ‘auto-conditioning’ and allow donor stem cell engraftment in the absence of chemotherapy. Patients who survive HSCT appear to do well in terms of immune reconstitution, regardless of what type of donor was used, with most patients achieving complete cellular and humoral recovery, are able to make vaccine responses and do not require immunoglobulin replacement [40]. In contrast, only about 50% of patients on long term ERT are able to discontinue immunoglobulin replacement therapy.

Less than 25% of patients with ADA-deficient SCID have a MSD or MFD available, and in such situations GT has become established as an accepted therapeutic option. Following initial development more than 20 years ago [41–43], GT for ADA-deficient SCID has become a milestone in medical advancement as the first European Union (EU) licensed ex vivo stem cell retroviral vector GT (Strimvelis™) [44, 45]. Initial approaches, prior to the development of Strimvelis™, utilised bone marrow or umbilical cord blood without preparative conditioning but resulted in inadequate ADA production and patients required ongoing ERT. Improvement of gene transfer methods and the introduction of non-myeloablative conditioning with low-dose busulfan prior to infusion to make space for the transfected cells resulted in effective immune reconstitution and, to date, no reports of genotoxic insertional mutagenesis [44, 46–48], in contradistinction to other primary immunodeficiencies treated by gene therapy using retroviral vectors [49–51]. The largest report to date by Cicalese et al. of 18 ADA-SCID patients treated with GT, with a median follow-up of 6.9 years, reported 100% survival with no leukemic transformations, reduced rate of infections and robust T-lymphocyte and later B- lymphocyte reconstitution, although the percentage of gene-corrected myeloid cells was much lower [44]. Advantages of GT include the absence of graft-versus-host disease risk, and faster initiation of therapy compared to that involved with a donor search when a MSD or MFD is not available. However, long-term outcome is not yet known and further monitoring is required to allow better understanding of the risks associated with GT compared to allogeneic HSCT or long term ERT. Although GT using gamma-retroviral vectors has demonstrated an excellent safety profile to date, new developments using lentiviral vector-mediated GT, which harnesses the potential to transduce both non-dividing and dividing cells, have been recently reported which display promising results in terms of both clinical efficacy and safety [52–54].

### Long term outcome

Transplantation using a MSD or MFD without conditioning early after diagnosis is associated with a good outcome in terms of survival and CD3+ recovery in the first year post HSCT [40], but little is known about the long term outcome and quality of immune reconstitution of patients with ADA-deficient SCID. Extended application of newborn screening for SCID may contribute to improving outcomes moreover in the future by allowing identification of infants with SCID (of all genetic causes) before the development of infections and other complications using the detection of T-cell receptor excision circles (TRECs). TRECs are pieces of DNA excised during development of the T-cell receptor, and are an accurate measure of thymic output. Patients with SCID have a significantly decreased number of TRECs which can be detected using the newborn dried blood spot [55]. Earlier diagnosis and a reduced burden of complications allows for more prompt intervention and improved outcome as studies indicate that siblings diagnosed based on a known family history have a significantly improved survival [56]. Despite the positive outlook in terms of immune reconstitution following HSCT as reported by Hassan et al., the follow-up period in this study was a maximum of 27.6 years (median 6.5 years) [40], and longer follow-up data are required to continue to evaluate the permanency of T-lymphocyte immunity, as initial data suggests that thymopoiesis is limited in unconditioned MFD/MSD HSCTs, which may lead to eventual exhaustion of the T-lymphocyte repertoire [40]. The prospects of GT as a therapeutic option are also promising; particularly with the development of refined vectors and gene editing technology, but further long term follow-up monitoring is needed. Development is also taking place in the improvement of PEG-ADA, with a clinical trial ongoing utilising a recombinant source enzyme as an alternative to the currently used bovine sequences [54]. Although no large prospective studies have been performed examining the outcome of non-immunological defects following definitive treatment, retrospective small reports suggest that neurological, behavioural and audiological defects are not corrected. Further studies are needed to examine if factors such as donor type and conditioning regimen, or type of therapy, influence outcomes in these areas. Further understanding is also needed of the underlying pathogenesis involved in the non-immune systemic manifestations to allow optimal investigation and management as well as to fully decipher between the metabolic effects of ADA-deficiency and effects inflicted by infectious agents.

## Conclusion

The ubiquitous expression of ADA means that deficiency can lead to a complex systemic metabolic disorder with multiple organ involvement with potential to cause significant morbidity unrelated to the immunodeficiency. Further understanding of non-immune manifestations is required. Early definitive therapy with HSCT using a MSD or MFD results in a good overall outcome, and GT is now an accepted therapeutic option for those without a suitable donor. The long term outcome of patients regardless of the type of therapy given is unknown and further monitoring is needed.

## Abbreviations

ADA: Adenosine deaminase
ATP: Adenosine triphosphate
dATP: Deoxyadenosine triphosphate
ERT: Enzyme replacement therapy
GT: Gene therapy
HSCT: Haematopoietic stem cell transplantation
MFD: Matched family donor
MMUD: Mismatched unrelated donor
MSD: Matched sibling donor
MUD: Matched unrelated donor
PAP: Pulmonary alveolar proteinosisis
PEG-ADA: Polyethylene glycol-conjugated adenosine deaminase
SAH: S-adenosylhomocysteine
SCID: Severe combined immunodeficiency
TCR: T cell receptor
TREC: T Cell receptor excision circle
